# A multifidelity approach coupling parameter space reduction and nonintrusive POD with application to structural optimization of passenger ship hulls

**DOI:** 10.1002/nme.7159

**Published:** 2022-11-15

**Authors:** Marco Tezzele, Lorenzo Fabris, Matteo Sidari, Mauro Sicchiero, Gianluigi Rozza

**Affiliations:** ^1^ Mathematics Area, mathLab SISSA, Scuola Internazionale Superiore di Studi Avanzati Trieste Italy; ^2^ Merchant Ships Business Unit Fincantieri S.p.A. Trieste Italy

**Keywords:** active subspaces, model order reduction, multifidelity, structural optimization, surrogate based optimization

## Abstract

Nowadays, the shipbuilding industry is facing a radical change toward solutions with a smaller environmental impact. This can be achieved with low emissions engines, optimized shape designs with lower wave resistance and noise generation, and by reducing the metal raw materials used during the manufacturing. This work focuses on the last aspect by presenting a complete structural optimization pipeline for modern passenger ship hulls which exploits advanced model order reduction techniques to reduce the dimensionality of both input parameters and outputs of interest. We introduce a novel approach which incorporates parameter space reduction through active subspaces into the proper orthogonal decomposition with interpolation method. This is done in a multi‐fidelity setting. We test the whole framework on a simplified model of a midship section and on the full model of a passenger ship, controlled by 20 and 16 parameters, respectively. We present a comprehensive error analysis and show the capabilities and usefulness of the methods especially during the preliminary design phase, finding new unconsidered designs while handling high dimensional parameterizations.

## 1 INTRODUCTION

When considering optimization of complex systems in an industrial context we must rely on surrogate models in order to alleviate the computational cost of this kind of many‐query problems.^1^, ^2^ Scientific machine learning^3^ is widely used in applied mathematics and in engineering applications^4^, ^5^, ^6^ such as inverse problems, optimization, and prediction of the behavior of parametrized systems, to cite a few. In this work we are considering structural reduced order models (ROMs) of passenger ship hulls in order to speed up the optimization process. Structural analysis of complex systems through reduced order modeling is not limited to naval engineering. Recently a component‐based data‐driven approach has been proposed to assess the structural integrity of aircraft components^7^, ^8^ in the context of modern digital twins incorporating not only data but also physical models, also referred to as hybrid twins.^9^ For multidisciplinary analysis and optimization involving reduction in both input and output spaces we cite References 10 and 11, while for a specific naval engineering application we suggest.^12^

In this work, we propose an optimization framework, to be used in the preliminary design phase, involving many reduced order models to assess the structural behavior of modern passenger ship hulls under different parametric configurations and loading conditions. Many studies have been conducted to assess the structural behavior of passenger ship hulls.^13^, ^14^, ^15^, ^16^ In Reference 17 they compare different surrogate models to improve the design process of complex thin‐walled ship structures, without using any proper orthogonal decomposition (POD)‐based model order reduction. For structural behavior and optimization of passenger ships we cite References 18 and 19 where they used efficient finite element modeling, evolutionary optimization algorithm and indirect constraint relaxation. We used a similar idea for the stability constraints, where local stress peaks are allowed to exceed the rule‐based strength limits.

A recent approach to accelerate PDE‐constrained optimization was proposed in Reference 20, where an adaptive method comprising both full order model evaluations and artificial neural networks surrogate models was used in the context of oil recovery. The main idea introduced was to use local approximations of the objective functional instead of a global surrogate model. Another adaptive numerical method involving ROMs and nonlinear trust‐region based on a residual error indicator able to keep the optimization trajectory consistent with the ROMs accuracy was presented in Reference 21.

The novelty of this work is the incorporation into the proper orthogonal decomposition framework of parameter space reduction^22^ by constructing a multi‐fidelity surrogate model,^23^, ^24^ without the need of running simplified simulations. This is done by exploiting the presence of an active subspace^25^ of the parameter to reduced state variables map. This represents a new data‐driven non‐intrusive ROM, more accurate with respect to a more classical interpolation method such as Gaussian process regression (GPR).^26^ We called this new method POD‐NARGPAS since it comprises POD with interpolation, nonlinear autoregressive Gaussian processes, and active subspaces. We also introduce a structural optimization numerical pipeline for large scale industrial applications, exploiting the newly proposed ROM. We use a Bayesian approach to perform discrete mono‐objective structural optimization. We incorporate stability constraints in a weak form by accounting for the added mass needed to stabilize the affected elements. The optimization pipeline, thanks to its modularity, allows for different target functions to minimize, from the total mass of the hull, to the manufacturing cost of the structure. Moreover, it has the potential of being used in many other engineering fields for surrogate‐based optimization tasks,^27^, ^28^ especially the ones involving costly numerical simulations.

In naval engineering multi‐fidelity methods have been used in the context of surrogate‐based design optimization for super‐cavitating hydrofoils,^29^ marine propellers design,^30^ and the optimization of a NACA hydrofoil with an adaptive sampling method using stochastic radial basis functions,^31^ for example.

This work is organized as follows: in Section 2 we present the entire pipeline; in Sections 3 and 4 we describe the full order model and the reduced order ones, respectively. We introduce parameter space reduction with active subspace, proper orthogonal decomposition with interpolation, and how to combine them in a multi‐fidelity autoregressive scheme. In Section 5 we briefly summarize the Bayesian optimization scheme we used for the numerical results reported in Section 6, where we tested the framework on a midship section of a simplified hull and on a real complete hull. Finally in Section 7 we draw the conclusions and some future research lines.

## 2 STRUCTURAL OPTIMIZATION PIPELINE

In this work, we use the Nested Analysis and Design (NAND)^32^, ^33^ approach. We consider the problem:

(1) minμ∈𝒫 f(s,μ),s.t.R(s,μ)=0,

where s represents the state vector, and R(s,μ) a general high‐dimensional discretized parametric partial differential equation (PDE), which we are going to characterize in Section 3. Following the NAND approach, where s is considered an implicit function of μ, we can rewrite the optimization problem as

(2) minμ∈𝒫 f(s(μ),μ).

So for every queried parameter point μ, we solve the PDE and evaluate the function to minimize. For a fast and accurate solution of the PDE we use reduced order models described in Section 4.

**FIGURE 1:**
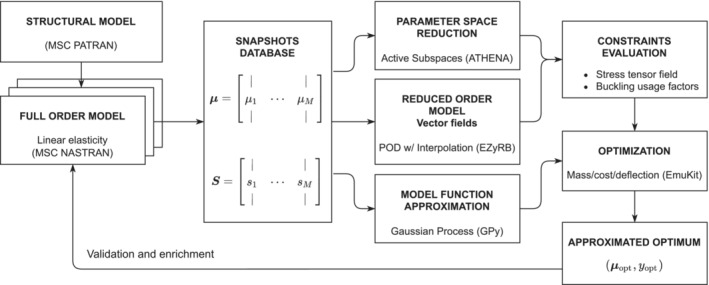
Structural optimization workflow, from the base structural model creation, to the approximated optimum and validation. Each block of the pipeline reports the underlying method and the software used.

The complete structural mono‐objective optimization workflow is depicted in Figure 1, in which for every building block we emphasize the software used.

We start from the construction of the parametrized structural model with MSC Patran, and we construct a database of full order solutions with MSC Nastran corresponding to a given set of parameters for every loading condition. With this database we construct different reduced order models depending on the quantity of interest we want to approximate. We use POD with interpolation (PODI)^34^, ^35^ for the stress tensor field approximation, and GPR^26^ for the approximation of scalar functions. Moreover we exploit active subspaces^25^ (AS) for the reduction of the parameter space dimension to build low‐fidelity models and improve the PODI prediction capabilities in a multi‐fidelity setting^24^, ^36^ called nonlinear autoregressive multi‐fidelity Gaussian process regression with active subspaces (NARGPAS).^23^ These parameter and model reduction methods are combined for a computational efficient and reliable evaluation of the constraints regarding the stability of the whole hull: we check how many elements are yielded, and how many elements are subjected to buckling phenomena. We remark that we allow local stress peaks to exceed the classification society rule limits, since we automatically incorporate within the function to optimize the necessary interventions at the shipyard to stabilize such elements. This is particularly important since the proposed pipeline is going to be used in the preliminary design phase. The optimization is done with a Bayesian approach. The approximated optimum is then validated with the full order model and the snapshots database enriched accordingly.

**FIGURE 2:**
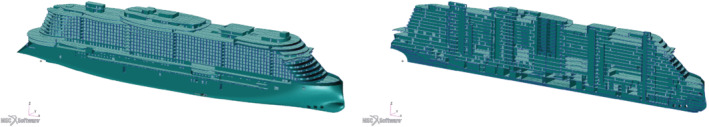
A complete view of the hull on the left, and a longitudinal section on the right

We are going to present all the numerical methods employed and finally the application of the whole pipeline to an actual passenger ship hull depicted in Figure 2 and built by Fincantieri S.p.A.

## 3 FULL ORDER MODEL

In this section, we describe the PDE we need to solve and the high‐fidelity solver used to create the solutions database.

The equations governing the linear elastic isotropic problem are the equilibrium equation, the linearized small‐displacement strain‐displacement relationship, and the Hooke's law, respectively:

(3) $$\left\{\begin{array}{ll} \; & -\nabla \cdot \sigma =h,\\ \; & \epsilon =\frac{1}{2}[\nabla u+\nabla u^{T}],\\ \; & \sigma =C(E,\nu ):\epsilon , \end{array}\right.$$

where σ is the Cauchy stress tensor, h is the body force, ϵ is the infinitesimal strain tensor, u is the displacement vector, and C is the fourth‐order stiffness tensor depending on E, the Young modulus, and on ν, the Poisson's ratio.

**FIGURE 3:**
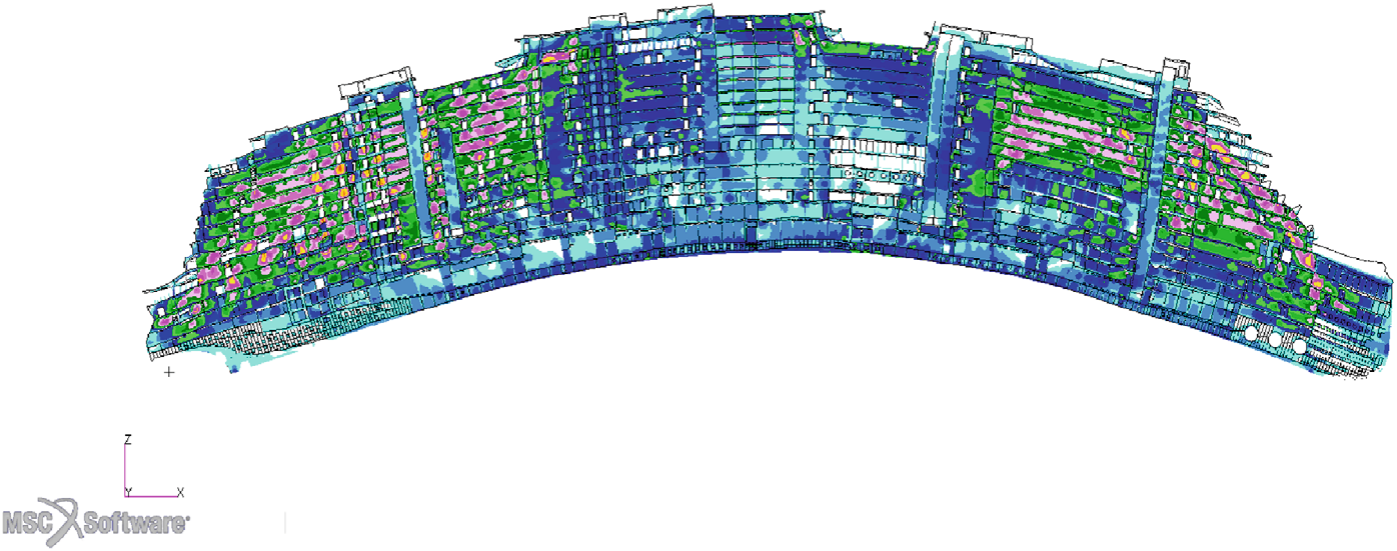
Possible deformation of the hull under the hogging loading condition. Displacements are magnified. Colors refer to the von Mises criterion

The finite element method employed uses 2‐dimensional elements, commonly referred to as plate and shell elements. They are used to represent areas in the model where one of the dimensions is small in comparison to the other two. The height or thickness of the element is substantially less than the width and the length. We are going to use MSC Nastran CQUAD4 and CTRIA3 elements, which are general‐purpose plate elements capable of carrying inplane force, bending forces, and transverse shear force. The membrane stiffness of the 2‐dimensional elements is calculated using the plane stress theory. Most thin structures constructed from common engineering material, such as aluminum and steel, can be modeled effectively using plane stress. In this work we consider only high strength structural steel (AH26) for illustrative reasons. The parameterization consists of the thickness associated to specific regions of the hull. We compute the solution for two classical loading conditions, namely the hogging and sagging. In Figure 3 an example of solution for the hogging loading condition. We are interested in the stress tensor field, through which we can compute the von Mises criterion and the buckling usage factors, used for the constraint's evaluation.

## 4 REDUCED ORDER MODELS

In this section, we describe the new proposed nonintrusive data‐driven ROM exploiting the low‐intrinsic dimensionality of the parameters to reduced snapshots map. In this work the parameters vector μ∈𝒫 represents the thickness of some selected macro areas of steel plates. In order to speed up the optimization procedure we construct a reduced order model for all the stress tensor components, and from them we compute the derived quantities which describe the constraints and the functions to optimize.

### 4.1 Sampling strategy

Since the steel plates can have only a finite set of possible thickness, the input parameter space is discrete. In order to cover the domain in a uniform unbiased way, we generate many random uniform sampling ${𝒫}^{i}:={\left\{\mu_{j}^{i}\right\}}_{j=1}^{M}$ composed by M samples each. We compute the minimum pairwise distance d associated to each ${𝒫}^{i}$ and we retain the samples with maximum d. Let us call 𝒫 the chosen samples set $𝒫:={\left\{\mu_{j}\right\}}_{j=1}^{M}$. This sampling plan allows to achieve univariate uniform distribution without manipulation of a Latin hypercube, while maximizing a space‐filling metric akin to the maximin Latin hypercube design technique from Reference 37. More sophisticated sampling schemes for discrete coordinates can be found in Reference 38.

### 4.2 Proper orthogonal decomposition with interpolation

Nonintrusive data‐driven POD‐based reduced order models are reviewed in Reference 39 with different applications. Here we briefly present POD with interpolation.

For every μ∈𝒫 we solve the associated high‐fidelity parametric problem defined above, and we store the solution snapshots $s_{j}:=s(\mu_{j})\in {\mathbb{R}}^{n}$, with j∈[1,…,M], in matrix form as follows:

(4) $$S=\left[\begin{array}{cccc} | & | & & |\\ s_{1} & s_{2} & \ldots & s_{M}\\ | & | & & | \end{array}\right].$$

We assume the state variables can be approximated as a linear combination of a few global basis functions, also called modes, that is

(5) $$s_{i}=\overset{M}{\underset{k=1}{\sum }}\varphi_{k}c_{i}^{k}\approx \overset{r}{\underset{k=1}{\sum }}\varphi_{k}c_{i}^{k},\;\forall i\in [1,\ldots ,M],\;\;r\ll M,$$

for some modes $\varphi_{k}\in {\mathbb{R}}^{n}$ and for some modal coefficients $c_{i}^{k}\in \mathbb{R}$. Equivalently in matrix form we have S≈ΦC, with $S\in {\mathbb{R}}^{n\times M}$, $\Phi \in {\mathbb{R}}^{n\times r}$, and $C\in {\mathbb{R}}^{r\times M}$. To compute such modes we use the proper orthogonal decomposition technique. We decompose the matrix S with the truncated singular value decomposition:

(6) $$S=U\sum V^{*}\approx U_{r}\sum_{r}V_{r}^{*},\;\;r\ll M,$$

where ${\;}^{*}$ denotes the conjugate transpose and the subscript r indicates the first r columns. The columns of U span the optimal low‐dimensional subspace in the least square sense and are also called POD modes. We have $\Phi :=U_{r}$. To compute the modal coefficients C, also called reduced state variables, we project the data onto the POD subspace: $C=\Phi^{T}S$.

With the matrices Φ and C we are able to reconstruct the initial database of solutions in a reduced way, but we are not able to predict the state vector corresponding to a new parameter $\mu^{*}$. In order to do so, we need to compute the parameters to reduced states map and then use the global basis to predict the entire stress field. We construct a function $g:𝒫\to {\mathbb{R}}^{r}$ which approximates the map $\mu \in 𝒫\to c\in {\mathbb{R}}^{r}$, given the initial set of M input‐output pairs ${\left\{\mu_{j},c_{j}\right\}}_{j=1}^{M}$, where $c_{j}=\Phi^{T}s(\mu_{j})$. The regression model g is used to predict the state $s^{*}=s(\mu^{*})$ by computing

(7) $$s^{*}=\Phi g(\mu^{*}).$$

This method is called POD with interpolation due to this regression function acting on the latent variables. Common choices for the interpolatory map are radial basis functions,^40^ Gaussian process,^41^, ^42^ cubic splines,^43^, ^44^ or artificial neural networks.^45^, ^46^ We are going to show how to compute an efficient approximation of such a map by exploiting only the directions of maximal variations in a multi‐fidelity setting, without the need to perform additional simulations.

### 4.3 Parameter space reduction through active subspaces

Active subspaces^25^ is a gradient‐based technique for parameter space reduction.^22^ Let $f:{\mathbb{R}}^{M}\to \mathbb{R}$ be a scalar function of interest. Through the eigendecomposition of the uncentered covariance matrix of the gradients, also denoted as the second moment matrix of ∇f, we can identify the direction of maximal variation of f along the parameter space. Let Q be such matrix:

(8) $$Q:={𝔼}_{\rho }\;[\nabla_{\mu }f\;\nabla_{\mu }f^{T}]=\int (\nabla_{\mu }f){\left(\nabla_{\mu }f\right)}^{T}\;\rho \;dℒ,$$

where ${𝔼}_{\rho }$ stands for the expected values with respect to the probability density function ρ, and dℒ is the Lebesgue measure. The function ρ characterizes the distribution of the input parameters. Q is symmetric positive definite and can be decomposed as $Q=W\Lambda W^{T}$. Analogously to what we have done for the POD, we retain the first $r^{\prime }\ll M$ eigenvectors, $W_{r^{\prime }}$, and we use them to project the data onto the so‐called active subspace, that is $\eta :=W_{r^{\prime }}^{T}\mu \in {\mathbb{R}}^{r^{\prime }}$. The truncation rank $r^{\prime }$ can be selected a priori or through the spectral decay of the matrix Q. With this projection we are essentially discarding the directions of the parameter space along which f is constant or almost constant. Finally we can construct a ridge approximation $g^{\prime }$ of the function of interest, that is

(9) $$f(\mu )\approx g^{\prime }(W_{r^{\prime }}^{T}\mu )=g^{\prime }(\eta ),$$

which lives on a low‐dimensional space.^47^ Usually a Gaussian process is used to construct such response surface. In the next section we are going to show how to exploit $g^{\prime }$ to build a low‐fidelity model and increase the accuracy of the reduced state predictions.

AS has been successfully used to approximate the POD modal coefficients under some assumptions on the size of the dataset.^48^ When in presence of different fidelity models for f a multi‐fidelity model for the computation of the AS can be used.^49^ Other applications in naval engineering can be found in References 50 and 51, while for coupling of AS with model order reduction methods see References 52 and 53.

### 4.4 Nonlinear autoregressive multi‐fidelity GP

The nonlinear autoregressive multi‐fidelity Gaussian process regression (NARGP) scheme was proposed in Reference 24. Let us consider the input/output pairs corresponding to p levels of increasing fidelity, that is

(10) $${𝒮}_{q}={\left\{x_{i}^{q},y_{i}^{q}\right\}}_{i=1}^{N_{q}}\subset 𝒳\times \mathbb{R}\subset {\mathbb{R}}^{M}\times \mathbb{R},\;\text{for}\;q\in \{1,\ldots ,p\},$$

where $y_{i}^{q}=f_{q}(x_{i}^{q})$, and q=1 stands for the lowest fidelity. Let $\pi :{\mathbb{R}}^{M}\times \mathbb{R}\to {\mathbb{R}}^{M}$ be the map projecting the data onto the first m coordinates corresponding to the input parameters. We assume the following hierarchical structure:

(11) $$\pi ({𝒮}_{p})\subset \pi ({𝒮}_{p-1})\subset \cdots \subset \pi ({𝒮}_{1}),$$

so that we only need a small number of high‐fidelity data with respect to the low‐fidelity ones. The hierarchy is due to the autoregressive nature of the method. The key step is to assign to each fidelity model $f_{q}$ a Gaussian process defined by the mean field $m_{q}$, and by the kernel $k_{q}$, as follows:

(12) $$y_{q}(\bar{x})-\epsilon ∼𝒢𝒫(f_{q}(\bar{x})|m_{q}(\bar{x}),k_{q}(\theta_{q}))\;\forall q\in \{1,\ldots ,p\},$$

where $\epsilon ∼𝒩(0,\sigma^{2})$ is a noise term and

(13) $$\bar{x}:=\left\{\begin{array}{ll} (x,f_{q-1}(x))\in {\mathbb{R}}^{d}\times \mathbb{R},\; & q>1\\ x\in {\mathbb{R}}^{d},\; & q=1 \end{array}\right..$$

The idea is to build a low‐fidelity model exploiting parameter space reduction through active subspaces. We are going to consider only two fidelity levels, where the lowest one is built without the need of new simulations coming from simplified models, but instead is a constant extension along the inactive subspace of the regression built along the active subspace. We call this approach NARGPAS. We use the high‐fidelity data as training set, following the algorithm presented in Reference 23, with the following multi‐fidelity model:

(14) $$g_{\text{MF}}=((f_{H}|x_{i}^{H},y_{i}^{H}),\;(f_{L}|x_{i}^{L}))∼(𝒢𝒫(f_{H}|m_{H},\sigma_{H}),𝒢𝒫(f_{L}|m_{L},\sigma_{L})),$$

where the H and L denote the high and low‐fidelity, respectively. The scalar quantity of interest we are going to model are the reduced state variables. The low‐fidelity is built by extending on the whole parameter space a one‐dimensional response surface constructed over the AS corresponding to each POD coefficient, thus the name POD‐NARGPAS. As we are going to show in the section devoted to the numerical results, the new proposed data‐driven approach outperforms the more classical single‐fidelity, without the need of any additional simulation. We remark that POD‐GPR, for naval engineering problems, has proven better than RBF and linear interpolation in Reference 42.

For the computation of the active subspace we used ATHENA^*^.^54^ For the construction of the reduced order models we used EZyRB†.^55^

## 5 BAYESIAN OPTIMIZATION

To minimize the model functions of interest we use Bayesian optimization,^56^, ^57^, ^58^ which we are going to briefly present in this section. It is a class of machine‐learning‐based derivative‐free global optimization methods. One of the main assumptions is that we do not have any information about the structure of the function to optimize, so it is intended as a black‐box. Bayesian optimization was first introduced in References 59, 60, 61, and successively made popular in Reference 62 in the context of efficient global optimization.

Mathematically, we are considering the problem of finding a global minimizer of an unknown function $f:\Omega \subset {\mathbb{R}}^{M}\to \mathbb{R}$, that is

(15) $$x_{\text{opt}}=\underset{x\in \Omega }{\text{arg min}}\;f(x),$$

where $\Omega \subset {\mathbb{R}}^{M}$ is the design space of interest, which in our case is the parameter space 𝒫. More general settings can include design spaces with less regularity due to the presence of possible nonlinear constraints. Here we consider a sequential search algorithm which selects the next location where to query f. The Bayesian posterior represents the best current knowledge of the function to optimize. The locations are selected by evaluating an acquisition function α:Ω→ℝ which leverages the uncertainty of the posterior to guide the exploration of the design space. In Algorithm 1 we sketch a pseudo‐code to highlight the main steps of the whole process. For the actual implementation we used the one provided by Emukit.^63^

Algorithm 1 **Bayesian optimization pseudo‐code**

1

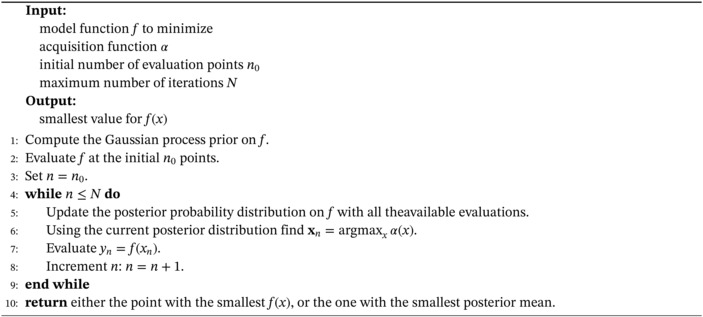

As acquisition function we adopt the expected improvement (EI), which is one of the most commonly used. Let

(16) $$f_{n}^{*}(x):=\underset{l\leq n}{\min}f(x_{l}),$$

be the value of the smallest observed values. We want to perform a new evaluation, say y=f(x), which has the highest expected improvement defined as:

(17) $$\alpha (x):={\text{EI}}_{n}(x):={𝔼}_{n}\left[{\left(f_{n}^{*}(x)-f(x)\right)}_{+}\right].$$

With ${𝔼}_{n}$ we denote the expectation taken under the posterior distribution given the first n evaluations, that is ${𝔼}_{n}[\cdot ]={𝔼}_{n}[\cdot |x_{1},\ldots ,x_{n},y_{1},\ldots ,y_{n}]$, while with ${\left(\cdot \right)}_{+}=\max(\cdot ,0)$ we denote the positive part. This acquisition function can be computed in closed form Reference 62. The actual next point $x_{n+1}$ to evaluate is then given by

(18) $$x_{n+1}=\arg\;\underset{x}{\max}{\text{EI}}_{n}(x).$$

To find it we exploit the simpler structure of α with respect to the target function f, which allows for inexpensive evaluations and also easy computation of first and second derivatives.

**FIGURE 4:**
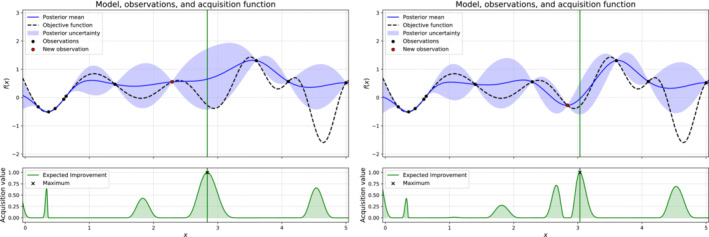
Illustration of the Bayesian optimization procedure for a given iteration (on the left) and the next iteration (on the right). The top part of the plots shows the estimated mean and confidence intervals of the unknown objective function (in dashed line). In the bottom part of the plots we show the acquisition function (in green) and its maximum.

In Figure 4 we show 2 iterations of a Bayesian optimization procedure for a test function, highlighting both the prediction and the value of the acquisition function. We notice that the acquisition is high where the model predicts a low objective (so‐called exploitation) and where the prediction uncertainty is high (so‐called exploration). Note that the area on the far left remains unsampled, as while it has high uncertainty, it is predicted to offer a smaller improvement over the best observation.

## 6 NUMERICAL RESULTS

In this section we are going to apply the optimization pipeline described in Section 2 to a midship section and to the parametrized hull depicted in Figure 2. The first test case is intended to be illustrative due to its smaller size and the possibility of showing all the error comparisons. We considered a midship section of a simplified hull with 20 parameters. It presents all the challenges of an entire hull in terms of stress distribution but with less degrees of freedom, allowing for faster high‐fidelity simulation. The second test case is a real passenger ship hull parametrized with 16 parameters, keeping the number of parameters comparable to the previous test. We perform a discrete mono‐objective optimization of the mass of the parametrized regions, considering stability constraints.

### 6.1 Objective function definition

The target function we are going to minimize is the total mass m of the parametrized regions of the hull plus the mass of the buckling stiffeners needed to stabilize the buckled elements of the entire hull. Other possible choices are available within the optimization framework such as the deflection at a selected point for a given loading condition, and the total cost related to the parametrized decks. The latter considers both the acquisition cost of the metal raw materials and the manufacturing cost for the installation of the steel plates and buckling stiffeners, specific for each shipyard. For industrial reasons in this work we are not going to present the results for the cost optimization, but we want to emphasize that the framework is very versatile and allows the use of different target functions.

As for the stability constraints we set some thresholds for the Cauchy stress tensor components in order to count how many plate elements are yielded for a prescribed set of loading conditions, which in the present work are hogging and sagging. See Figure 3 for an example of hogging condition. Given the symmetric Cauchy stress tensor in the global reference frame, whose components for a single element are

(19) $$\sigma =\left[\begin{array}{ccc} \sigma_{x} & \tau_{xy} & \tau_{xz}\\ \tau_{xy} & \sigma_{y} & \tau_{yz}\\ \tau_{xz} & \tau_{yz} & \sigma_{z}, \end{array}\right],$$

we define an element yielded if at least one of the following conditions is not satisfied:

(20) $$\begin{array}{ll} -\;245 & \leq \sigma_{i}\leq 245,\;\text{for}\;i\in \{x,y,z\}, \end{array}$$

(21) $$\begin{array}{ll} -\;153 & \leq \tau_{i}\leq 153,\;\text{for}\;i\in \{xy,xz,yz\}, \end{array}$$

(22) $$\begin{array}{ll} \sigma_{\text{VM}} & :=\sqrt{\sigma_{x}^{2}+\sigma_{y}^{2}-\sigma_{x}\sigma_{y}+3\tau_{xy}^{2}}\leq 307, \end{array}$$

where $\sigma_{\text{VM}}$ stands for the von Mises yield criterion. The thresholds above are characteristics of the high strength structural steel. The actual constraint for the optimization is the maximum number $N_{\max}^{y}$ of elements that can yield. Exactly the same is done for the buckling usage factors associated to each element. An element is considered buckled, based on the DNV GL classification rules^‡^, if at least one of the 11 components of the buckling usage factors tensor is greater than 1, for at least one loading condition. Such tensor is computed as a function of the Cauchy stress tensor. The maximum number of allowed buckled elements is $N_{\max}^{b}$. To incorporate these stability constraints we penalize the objective function $f_{\text{obj}}$ with a parabolic function depending on the violated constraint. Its expression is the following

(23) $$f_{\text{obj}}(\mu ):=m(\mu )+m_{\text{bs}}N^{b}(\mu )+c_{y}{\left(N^{y}(\mu )-N_{\max}^{y}\right)}_{+}^{2}+c_{b}{\left(N^{b}(\mu )-N_{\max}^{b}\right)}_{+}^{2},$$

where m(μ) is the mass of the parametric decks, $N^{y}(\cdot )$ and $N^{b}(\cdot )$ denote the number of yielded and buckled elements, respectively, $m_{\text{bs}}=2.968$ kg is the mass of a single buckling stiffener, $c_{y}=1$, $c_{b}=0.001$, and ${\left(\cdot \right)}_{+}=\max(\cdot ,0)$ stands for the positive part. The coefficients $c_{y}$ and $c_{b}$ are prescribed by the user depending on the order of magnitude of the other terms. With this formulation we allow local stress peaks to exceed the rule‐based strength limits because we are able to account for the additional mass needed to stabilize the affected elements.

### 6.2 Midship section

**FIGURE 5:**
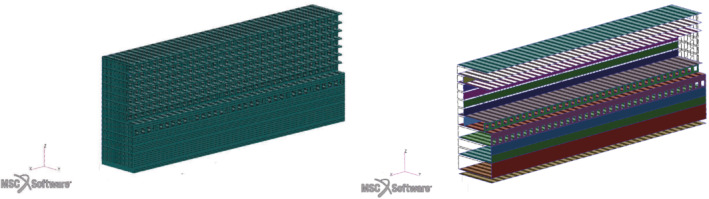
A complete view of the midship section on the left, and the highlight of the 20 parametrized elements groups on the right

For the testing and tuning phases of the pipeline development, we consider a smaller model to obtain faster high‐fidelity evaluations. This midship section, shown in the left panel of Figure 5, approximates a quarter of the ship's elements, excluding the bow and stern segments; the finite element problem applies mirroring along both negative x and negative y coordinates, as the origin represent the ship's center. Due to this simplification and the very regular internal structure, this midship section contains about $\frac{1}{10}$ of the elements of the full ship and the required high‐fidelity solve time is about $\frac{1}{20}$.

The parametrized sections, depicted in the right panel of Figure 5, were chosen with the aim of studying the structural resilience against longitudinal loads: all groups of elements have the largest span along the x direction. Of these, the 9 decks included are equally distributed between the lowermost, uppermost and central; whereas the groups which span vertically comprise the 4 external plankings between decks 1 and 5, the 6 internal twin decks between decks 5 and 11, and finally the external twin deck between decks 5 and 6. Table 1 describes the actual parameter space $𝒫\subset {\mathbb{R}}^{20}$ used, the location of the area affected by a specific parameter and its default value.

**TABLE 1:** Parameters description of the hull for the midship test case

Parameter	Region	Default thickness	Lower bound	Upper bound
$\mu_{1}$	Deck 12	6.0	5.0	20.0
$\mu_{2}$	Deck 11	6.0	5.0	20.0
$\mu_{3}$	Deck 10	5.5	5.0	15.0
$\mu_{4}$	Twin deck 5,6	5.0	5.0	15.0
$\mu_{5}$	Twin deck 6,7	5.0	5.0	15.0
$\mu_{6}$	Twin deck 7,8	5.0	5.0	15.0
$\mu_{7}$	Bottom	14.0	12.0	20.0
$\mu_{8}$	Deck 1	13.0	12.0	20.0
$\mu_{9}$	Deck 2	6.0	5.0	15.0
$\mu_{10}$	Twin deck 8,9	5.0	5.0	15.0
$\mu_{11}$	Twin deck 9,10	5.0	5.0	15.0
$\mu_{12}$	Twin deck 10,11	5.0	5.0	15.0
$\mu_{13}$	Deck 4	5.0	5.0	15.0
$\mu_{14}$	Deck 5	5.0	5.0	15.0
$\mu_{15}$	Deck 6	5.0	5.0	15.0
$\mu_{16}$	External twin deck 5,6	8.0	5.0	15.0
$\mu_{17}$	External planking 4,5	10.0	8.0	15.0
$\mu_{18}$	External planking 3,4	10.0	8.0	15.0
$\mu_{19}$	External planking 2,3	12.0	8.0	15.0
$\mu_{20}$	External planking 1,2	12.0	8.0	15.0

*Note*: All data are in mm.

**FIGURE 6:**
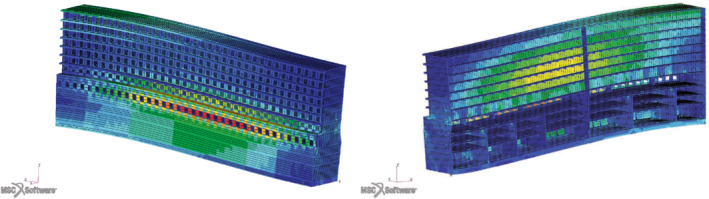
The external view of the sagging midship section on the left, and the internal view of the same configuration on the right. Displacements are magnified. Colors refer to the von Mises criterion

In Figure 6 the von Mises stress values are shown under sagging. The effect of shear stresses on the external plankings and twin decks is particularly pronounced on the central twin decks, as well as the normal stresses on the central and uppermost decks. These images were obtained for the default parameters configuration, where the mentioned groups are very thin.

**FIGURE 7:**
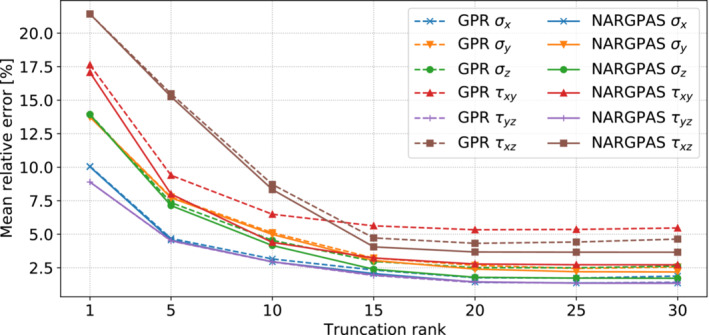
The mean relative $L^{2}$ error of the stress field varying the truncation rank, for GPR (dashed lines) and NARGPAS (continuous lines) interpolators. For each feature, the values are obtained by averaging the test scores from a 5‐fold cross‐validation experiment.

We consider an initial database of 300 high‐fidelity solutions, and we adopt a 5‐fold cross validation scheme in order to estimate the prediction errors for the stress tensor components. In Figure 7 we compare the mean relative $L^{2}$ errors of each component, obtained with GPR and NARGPAS interpolators for different POD truncation ranks. The general trend is a reduction of the errors as the truncation rank increases, with NARGPAS achieving better error values than GPR especially for $\tau_{xy}$ with a 50% reduction.

**FIGURE 8:**
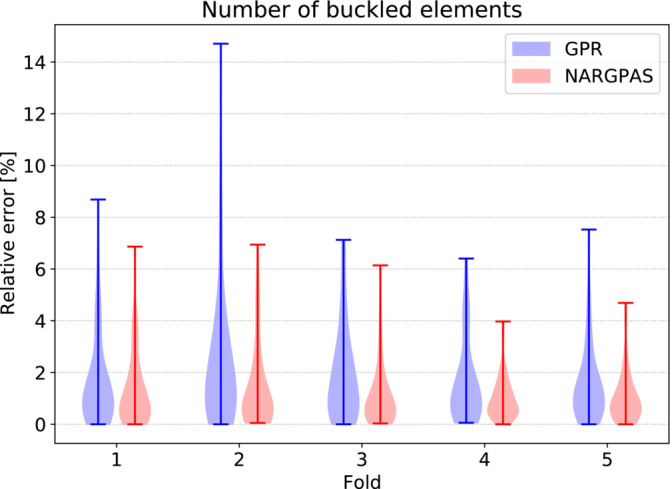
Violin plot describing the distribution of the relative error in the prediction of the number of buckled elements. Data are divided by fold and methods used.

In Figure 8, we compare the distribution of the relative prediction errors on the number of buckled elements in each fold of the cross validation experiment. As expected from the stress tensor components prediction errors, NARGPAS outperforms GPR in every fold and achieves both lower maxima and lower spread, presenting a greater accumulation of the errors near 0. The POD‐NARGPAS approach in general provides better perfoamnce both in the approximation of the stress tensor field and to the derived quantities of interest related to the stability of the hull. The results regarding the number of yielded elements are not reported since already with the POD‐GPR method the predictions are very close to the actual values and the differences are negligible.

After constructing a reduced order model for every component of the stress tensor we can perform the Bayesian optimization described above. The truncation rank for the stress components is 21, chosen by analyzing the singular values decay of the snapshot matrix and selecting the rank r which manifests the last sharpest decrease between r and r+1, among all the stress tensor components. The chosen rank results in a cumulative energy greater than 99.99%. We emphasize that every parametric term in subsection 23, except m(μ), is predicted using the precomputed reduced order models. In this way a single parameter evaluation takes approximately 1 second. The term m(μ) is computed exactly. For this case we set $N_{\max}^{y}=20$ and $N_{\max}^{b}=4000$.

**FIGURE 9:**
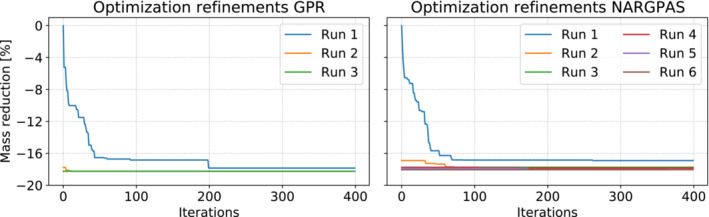
Different optimizations runs for the midship test case. On the left panel we used the POD‐GPR approach, while on the right panel the POD‐NARGPAS one. The relative reduction is with respect to the best sample among the initial solutions database.

We set the computational budget for the Bayesian optimization to 400 iterations. We remark that at every iteration the GPR for the target function has to be recomputed. This means that the GPR construction time increases at every iteration. In Figure 9 the results for all the successive optimization runs are depicted. After an optimization cycle is completed, a subset of the best low‐fidelity configurations evaluated is sent to the high‐fidelity solver and the results are added to the snapshots database. The reduced order models are then updated. Thus the accuracy in the neighborhood of the current optimum is increased and the successive runs will exploit such information by focusing in that specific region, or will explore different areas if the error committed at the current optimum is too high. For this test case at every enrichment we add 20 high‐fidelity evaluations. We continue to perform optimization runs followed by the enrichment phase until the optimizer is not able to find a better point with respect to the previous run. The main source of errors is in counting the buckling stiffeners needed to stabilize the buckled elements as can be observed looking at subsection 23 and at the previous plots.

The hull configurations found by the algorithm using GPR and NARGPAS, although with small differences, modified the default hull in the same way. In both, the external planking was consistently slimmed down, together with all decks except the central ones and the uppermost, which were instead left unchanged. All twin decks were slightly bulked up, except for the external one which was slimmed down. The final mass for the configuration found by the GPR interpolator is only 0.23% lower than that obtained with NARGPAS, as can be seen in Figure 9. Since both configurations acted on the default parameter values in the same way, the algorithm appears robust.

**FIGURE 10:**
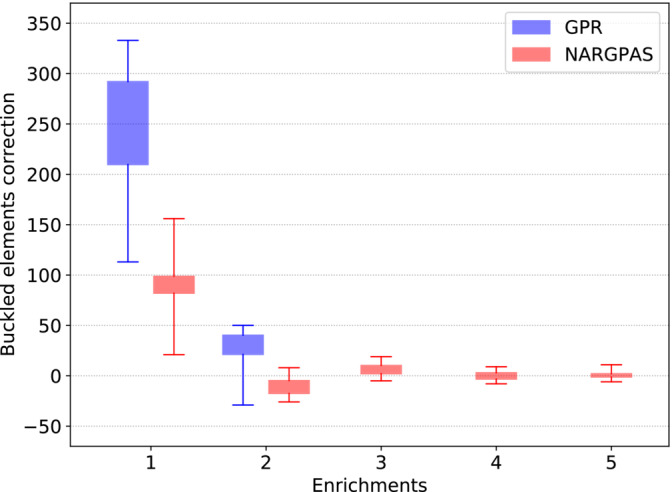
Evolution of the prediction error distribution of the number of buckled elements for the validated best candidates, after every optimization‐enrichment loop.

The optimization of GPR‐based surrogates ended with the third iteration, while the NARGPAS‐based ones required six runs. In Figure 10 we summarize the evolution of the prediction error distribution on the optimal hulls during the optimization‐enrichment loop. After each round of Bayesian optimization, the best 20 hull configurations are selected for the enrichment. For each one, the difference between the number of buckled elements predicted by the ROMs used in the previous optimization run and its high‐fidelity counterpart are stored and the resulting distributions form the boxes and whiskers. For both interpolators, the error is largest at the first iteration and decreases as the high fidelity database is enriched. GPR‐based surrogates exhibit larger errors compared to NARGPAS, which despite having required a larger number of iterations, seem to provide a faster convergence of the error.

### 6.3 Complete hull

**FIGURE 11:**
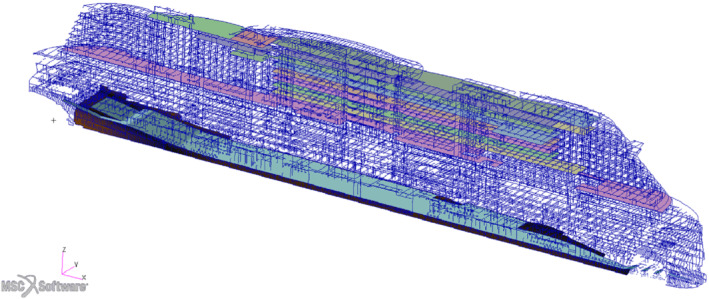
View of the hull parametric decks. In solid colors the regions of the hull affected by the parameters described in Table 2.

For the second test case we decreased the number of input design parameters to 16, but used a larger and more complex model. The parametric regions are depicted with solid colors in the right panel of Figure 11, while a deeper description of the range of variations of each parameter $\mu_{i}$, i=1,…,16, can be found in Table 2. We use the sampling strategy described in Section 4.1 to generate 300 samples. The truncation rank for the stress components is set to 17, for which the cumulative energy of the singular values is greater than 99.99%. We also increase the computational budget for the Bayesian optimization to 600 evaluations. During the enrichment phase we evaluate the best 4 hull configurations found by the optimizer.

**TABLE 2:** Parameters description for the entire hull

Parameter	Region	Default thickness	Lower bound	Upper bound
$\mu_{1}$	Deck 15	7.5	5.0	15.0
$\mu_{2}$	Deck 16	8.0	5.0	20.0
$\mu_{3}$	Deck 17	9.0	5.0	20.0
$\mu_{4}$	Deck 14	7.5	5.0	15.0
$\mu_{5}$	Deck 13	7.0	5.0	15.0
$\mu_{6}$	Deck 12	6.5	5.0	15.0
$\mu_{7}$	Deck 11	6.0	5.0	15.0
$\mu_{8}$	Deck 10	5.5	5.0	15.0
$\mu_{9}$	Deck 17	6.0	5.0	20.0
$\mu_{10}$	Deck 17	15.0	5.0	20.0
$\mu_{11}$	Deck 17	6.0	5.0	20.0
$\mu_{12}$	Deck 16	6.0	5.0	20.0
$\mu_{13}$	Deck 16	6.0	5.0	20.0
$\mu_{14}$	Deck 09	8.0	5.0	15.0
$\mu_{15}$	Deck 01	16.0	12.0	25.0
$\mu_{16}$	Deck 00	20.0	12.0	25.0

*Note*: Repeated regions correspond to different parts. All data are in mm.

**FIGURE 12:**
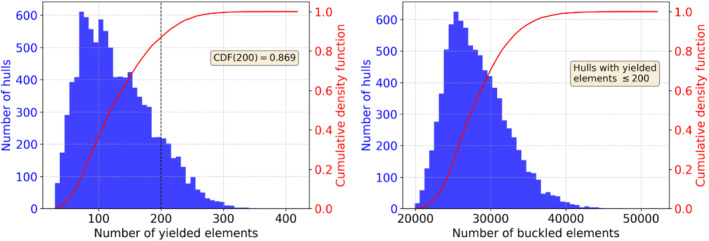
Distribution of the hulls with respect to the stability constraints. In the left panel we consider the number of yielded elements, while in the right panel we have the distribution of the parametric hulls satisfying the first constraint with respect to the number of buckled elements.

To have a better idea on how to set the stability constraints for a given type of hull, we can plot the distribution of the parametric hulls with respect to the number of yielded elements (left panel of Figure 12). We see that if we set $N_{\max}^{y}=200$, the valid samples will be roughly 87% of all the manufacturable hulls. In the right panel of Figure 12 we plot the distribution of the hulls satisfying such constraint with respect to the number of buckled elements and we set $N_{\max}^{b}=35000$ accordingly.

**FIGURE 13:**
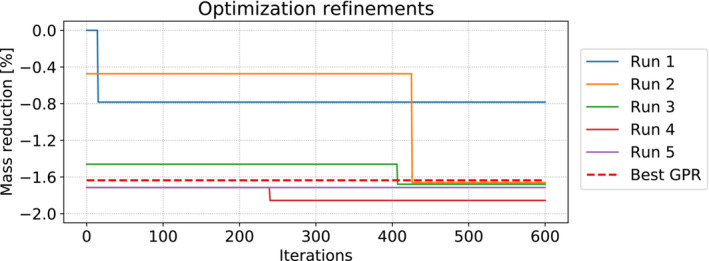
Different optimizations runs for the parametrized hull test case. The relative reduction is with respect to the best sample among the initial solutions database.

Figure 13 depicts the successive runs performed using the NARGPAS method, compared with the best value found using GPR to predict the reduced state variables. We need 5 optimization‐enrichment loops to achieve global convergence in the approximated solution manifold. As we can see the fifth run does not produce any new improvement. In particular from the figure we conclude that the third run produces the actual optimum. The slight difference is due to the approximation error in the computation of the number of buckled elements, which is not present in the successive run since that snapshot has been added to the database.

The configurations found by the two optimization loops differ more than in the midship section case. Both agree on choosing much thinner bottom decks and bulking up the mid deck. However, the upper decks, which are divided into multiple sections, show disagreement between the two with how the default parameter values are changed. Nonetheless, the final mass scores only differ by 0.08%. Absolute values are not reported for industrial reasons. Even if the mass reduction seems not as remarkable, this is due to the fact that the reference hull, present in the initial database, was obtained after several iterations of the design office and actually manufactured. With this test case we can see how our framework is able to find better designs while preserving the stability constraints in a non‐intrusive fashion. From the designers' point of view, the changes on the lower and mid decks are in line with the professional experience accumulated in years of work spent hand‐tuning the initial default parameter choices. The application of said experience, however, still requires the designer to proceed with trial and error, which translates to many days of work due to the long computation time of the high‐fidelity solver and the results analysis. The availability of an automated procedure which can bootstrap this process in mere hours, and requires minimal input from the user, speeds up the design phase by a large margin.

## 7 CONCLUSIONS AND PERSPECTIVES

In this work we proposed a modular data‐driven non‐intrusive structural optimization framework for modern passenger ships. We exploit several reduced order models coupled with parameter space reduction in a multi‐fidelity setting. This new approach is called POD‐NARGPAS. We demonstrated its performance against the more classical POD‐GPR. Our efficient numerical pipeline allows for different discrete mono‐objective optimization given a precomputed set of high‐fidelity simulations. We parametrized the thickness of various regions of the reference hull and we perform a mass minimization considering stability constraints such as the total number of yielded and buckled elements. We allow the presence of unstable elements since we are able to account for all the necessary interventions to stabilize them during the optimization process. We provided a comprehensive error analysis for a midship section test case which presents all the challenges of a complete hull while keeping low the time to solution. Finally we tested the entire framework on a real cruise ship and show how the tool is able to find previously unconsidered designs.

Future works will focus on improving the accuracy of constraints evaluations, for example with a multi‐fidelity approximation of the scalar output and not only for the reconstruction of the entire field.^23^ Another possibility is the exploitation of local information with local active subspaces^64^ or nonlinear techniques, based on kernels^65^ or level‐sets,^66^, ^67^ to further improve the regression performance of the low‐fidelity model. Other physical constraints can also be considered such as the position of the center of mass. Regarding the optimization procedure, a natural evolution is the implementation of a multi‐objective optimization which considers at the same time both mass and deflection at a given point, for example. This can be done in a Bayesian setting, accounting for high dimensional input parameter space,^68^, ^69^ but also other approaches should be considered, such as genetic algorithms enhanced by active subspaces.^70^, ^71^

## CONFLICT OF INTEREST

The authors declare no potential conflict of interests.

## Data Availability

The data that support the findings of this study are available on request from the corresponding author. The data are not publicly available due to privacy or ethical restrictions.

## ENDNOTES

* Available at https://github.com/mathLab/ATHENA.

† Available at https://github.com/mathLab/EZyRB.

‡ Det Norske Veritas (DNV) Rules for Ships, part 3, chapter 1, section 13: Buckling control.
